# Occupational health education and training as a sustainable development goal

**DOI:** 10.3389/fpubh.2026.1787794

**Published:** 2026-04-29

**Authors:** Johannes Siegrist, Ulrike Bollmann, Dingani Moyo

**Affiliations:** 1 Centre for Health and Society, Faculty of Medicine, Heinrich-Heine-University Düsseldorf, Düsseldorf, Germany; 2 European Network Education and Training in Occupational Safety and Health (ENETOSH), Dresden, Germany; 3 School of Public Health, University of the Witwatersrand, Johannesburg, South Africa; 4 Department of Community Medicine, National University of Science and Technology, Bulawayo, Zimbabwe

**Keywords:** academic training, African universities, decent work, occupational health, SDG 8

## Introduction

The 17 Sustainable Developmental Goals (SDGs) declared by the United Nations (UN) in 2015 represent a global challenge to public health toward achieving a viable future of human life, requiring substantial efforts of research, education, and national as well as international policies (1). Goal Number 8 (SDG 8) was intended to promote sustained, inclusive, and sustainable economic growth, full and productive employment, and decent work for all. To promote worldwide decent work is considered a particularly ambitious goal, given the globally far-reaching poor, health-adverse working and employment conditions of working populations (2). According to a program-assessment by the UN, SDG 8 belongs to a small number of these 17 goals with least progress achieved so far (3). Strengthening occupational health and safety by enforcing protective national policies and by providing well-trained professional services has been shown to substantially improve decent work in developed countries (4). It is therefore expected that investments into professional occupational health and safety training at universities offer a promising complementary step toward advancing SDG 8 (5).

To explore some evidence in favor of this assumption, we conducted a main online survey among academic leaders of university programs in Occupational Medicine (OM), Occupational Safety, Industrial Hygiene and Ergonomics (OSI), and Human Resource Management (HRM). The online survey included ninety universities recruited from 29 European countries. In view of compelling challenges of decent work in the Global South, we performed an additional preliminary survey with leaders of 12 study programs in nine universities recruited from seven African countries. The findings of this latter, much smaller study reflect a preliminary assessment, whose results deserve to be followed up in the future. In both studies, we were interested to learn to what extent main topical content related to SDG 8 was implemented in these programs and which core learning objectives guided this training. Moreover, the practical impact of these programs on worksite health promotion within and beyond universities was analyzed. The studies were approved by the ethical committees of respective organizations. Here, we provide a short summary of the main findings of these investigations, and we critically discuss a potential role of improved academic occupational health and safety training in advancing decent work at a global scale.

## Two exploratory analyses of the role of academic training in promoting SDG 8

The European online survey included 57 OSI, 37 OM, and 20 HRM programs (6) (Table 1). Program leaders, in most instances university professors, were identified via national professional associations that distributed the online questionnaire to their members. Neither the proportion of distributed questionnaires by national associations nor the names of persons contacted, due to data protection regulations, could be identified, thus preventing the calculation of response rates. With this distributional process, it is likely that countries and universities with more advanced academic developments were overrepresented in the sample. To answer the first question concerning SDG 8-related content, four topics were proposed for its description, based on the authors' preliminary screening of curricular content: economic globalization and climate change; social inequalities in health; new work, digitization and precariousness; and work-related diseases and their prevention. The relative importance of these topics was rated, and the frequency of included topics in a respective program was documented. Not surprisingly, OSI and OM programs put their main emphasis on “work-related diseases and their prevention,” mentioned most often, whereas HRM programs rated the first two topics as most important ones. However, overall coverage of these topics within all programs was rather limited. Topics with highest frequency were reported in only 41 percent of OSI, 39 percent of OM and 32 percent of HRM programs. As a result, SDG 8-related content was included to a very limited extent only in these programs, specifically in the training of future occupational physicians and safety professionals. As core learning objectives, knowledge transmission and critical thinking ranked highest in all three types of programs, whereas a focus on ethical values and on the development of socio-emotional and technical skills received less interest. Lecturing by senior faculty members was the most common didactical approach, while student-led teaching or experiential learning were rarely mentioned. Finally, the practical impact of these training programs on worksite health promotion was rather modest, as most activities were directed toward health improvements within the university's organizational context.

**Table 1:** Overview of academic study programs in the two study regions.

European programs			African programs		
Program type	No of countries	*N*	Program type	No of countries	*N*
OSI	29	57	OSI	9	^*^
OM	29	37	OM	9	5
HRM	4	20	HRM	4	7

^*^Occupational Safety and Industrial Hygiene and Ergonomics programs (OSI) were partly integrated into Occupational Medicine programs (OM).

The 90 European universities offered 114 programs; the nine African universities offered 12 programs (6).

The African study was developed in collaboration with African members of the International Commission on Occupational Health (ICOH) and of the Occupational Safety and Health Africa Foundation (OSH Africa), with the selection criteria of representing each one of four subregions (except North Africa) and of offering OM, OSI or HR graduate and postgraduate programs (7). Through recommendations nine out of 11 invited universities collaborated, representing twelve study programs in seven countries (Botswana, DR Congo, Ghana, Kenya, Nigeria, South Africa, and Zimbabwe). Semi-structured interviews were conducted with program directors, mainly university professors, with identical wording of the study questions, thus enabling the comparison of answers. Five OM/OSI programs and four HRM programs are offered at master or postgraduate level, three HRM programs at MBA or bachelor level. Our report is restricted to the nine programs at master and postgraduate level. The findings from this study differed quite remarkably from the ones obtained in Europe. Importantly, all OM and OSI programs considered “economic globalization and climate change” and “social inequalities in health” as most important topics of their courses, often linked to prevailing local challenges, such as hazardous work in mining or mental health threats to informally employed workers. In HRM programs, initiatives of developing green economy were prioritized, and two innovative master programs were recently implemented to prevent environmental degradation and its adverse health effects, such as heat stress and flooding. Compared to the European study, ethical problems were more often rated as very important, and practical field work as well as project-based learning with active involvement of students were more common. We also observed that hybrid teaching was more widely implemented in the African context. Concerning practical impact of training programs, a higher amount of direct transfer from academy to worksites was observed, reflecting the shortage of a qualified occupational health and safety workforce in these countries.

Implementation of measures to improve physical and mental health in mining companies, initiatives of strengthening the health of working women, and participation in government expert committees on occupational health are examples of a direct transfer of outcomes from student projects and fieldwork.

When comparing the two reports, it is evident that SDG 8 was rarely judged an explicit and challenging “hot” topic within the European study programs of occupational health and safety under consideration. Priority was given to the transmission of expertise on traditional core content of occupational health hazards and their prevention. In contrast, SDG 8, linked to its global and social challenges, is firmly anchored in the African university programs examined, not only as teaching content, but also as a practical challenge. Academic leaders demonstrate a high level of awareness of economic and environmental developments, and they closely link academic education with social engagement. These differences may reflect the fact that immediate threats of globalization and climate change are witnessed more intensely in the Global South, while, at the same time, resources of occupational health services and legal protection policies are much more deficient than in the Global North (8, 9).

## Discussion

In our opinion, universities, through their educational and training programs, play an important role in advancing SDG 8, given their mission as main drivers of social progress and as agencies shaping knowledge and competences of future generations of leaders (4). Given the findings of the European survey, it seems that academic training programs of future occupational health and safety professionals and of human resource managers in this region were not sufficiently addressing newly arising problems of health at work in a rapidly changing, globally transformed world of work and employment, as those emphasized in SDG 8. While their main interest in controlling and securing national legal requirements of occupational safety and health is well-appreciated, this concern may prevent their pro-active coping with challenges of globally rising social inequalities in work-related health and of macro-structural threats to human welfare in a context of dramatic technological change and psychosocial uncertainty (10, 11). It appears from our preliminary results that the African study programs are more open to these latter challenges.

Admittedly, in view of critical macro-level developments, such as the extension, rather than reduction of informal work, a decline of global compliance with freedom of associations and collective bargaining rights, reduced investments into protective policies and occupational health and safety resources, and the devastating effects of economic pressure and competition on workers' health and wellbeing (2, 11), educational efforts of improving pro-active professional competences may be overridden. Yet, we may look at recent achievements of promoting decent work by strengthening national labor and social policies and by extending training and provision of services of occupational health and safety professionals in several countries. Importantly, these countries are not restricted to the Global North, such as the Scandinavian region (12) or Canada (13), but they include the Asian-Pacific region (14) as well. Strengthening these latter developments and striving toward their extension by policy initiatives is still considered an option of action in times of increasing threats.

In conclusion, our exploratory analysis of implementation of the SDG 8 Goal into academic training programs of occupational health and safety in two regions, universities in 29 European countries and universities in seven countries of Africa, documented interesting differences between these two regions, with more initiatives observed in the African context. This finding points to further developmental needs in adapting academic programs of occupational health to the challenges of a globally transformed world of work and employment.
